# Tribo-Mechanical Properties of the Antimicrobial Low-Density Polyethylene (LDPE) Nanocomposite with Hybrid ZnO–Vermiculite–Chlorhexidine Nanofillers

**DOI:** 10.3390/polym12122811

**Published:** 2020-11-27

**Authors:** Karla Čech Barabaszová, Sylva Holešová, Marianna Hundáková, Alena Kalendová

**Affiliations:** 1 Nanotechnology Centre, VŠB-Technical University of Ostrava, 17. listopadu 15/2172, 708 00 Ostrava-Poruba, Czech Republic; sylva.holesova@vsb.cz (S.H.); marianna.hundakova@vsb.cz (M.H.); 2 Department of Polymer Engineering, Faculty of Technology, Tomas Bata University in Zlín, Vavrečkova 275, 760 01 Zlín, Czech Republic; kalendova@utb.cz

**Keywords:** LDPE nanocomposites, antimicrobial hybrid nanofillers, structural phase characterization, tribo-mechanical properties, wear resistance

## Abstract

Materials made from low-density polyethylene (LDPE) in the form of packages or catheters are currently commonly applied medical devices. Antimicrobial LDPE nanocomposite materials with two types of nanofillers, zinc oxide/vermiculite (ZnO/V) and zinc oxide/vermiculite_chlorhexidine (ZnO/V_CH), were prepared by a melt-compounded procedure to enrich their controllable antimicrobial, microstructural, topographical and tribo-mechanical properties. X-ray diffraction (XRD) analysis and Fourier transform infrared spectroscopy (FTIR) revealed that the ZnO/V and ZnO/V_CH nanofillers and LDPE interacted well with each other. The influence of the nanofiller concentrations on the LDPE nanocomposite surface changes was studied through scanning electron microscopy (SEM), and the surface topology and roughness were studied using atomic force microscopy (AFM). The effect of the ZnO/V nanofiller on the increase in indentation hardness (HIT) was evaluated by AFM measurements and the Vickers microhardness (HV), which showed that as the concentration of the ZnO/V nanofiller increased, these values decreased. The ZnO/V and ZnO/V_CH nanofillers, regardless of the concentration in the LDPE matrix, slightly increased the average values of the friction coefficient (COF). The abrasion depths of the wear indicated that the LDPE_ZnO/V nanocomposite plates exhibited better wear resistance than LDPE_ZnO/V_CH. Higher HV and HIT microhardness values were measured for both nanofillers than the natural LDPE nanocomposite plate. Very positive antimicrobial activity against *S. aureus* and *P. aeruginosa* after 72 h was found for both nanofiller types.

## 1. Introduction

Low-density polyethylene (LDPE) is an engineering thermoplastic polymer that plays an eminent role in various industrial branches, especially in the medical field and biomedical industry. LDPE is the base material for artificial heart valves, stents, blood bags, catheter tubing etc. [1]. A key reason for the selection of LDPE in applications is LDPE’s comparatively inexpensive production process, easy processing (ease of moulding), excellent durability and non-toxicity and, of course, the low price of the final LDPE products.

Increasing requirements for materials for medical and biomedical applications have led to the development of new materials (such as nanocomposite materials) with specific surface properties. Emphasis is currently placed on the immediate and long-term antibacterial (or antimicrobial) nature of the (nano)materials; biocompatibility and bioactivity, including the cell and hemocompatibility of the (nano)materials; a low surface energy; and a high hydrophobicity, leading to poor wettability and poor adhesion [2,3]. Especially, the surface properties are modified by various surface treatment techniques such as plasma polymerization [4], UV-induced polymerization [5] or ozone treatment [2,6,7]. However, in the case of polymeric nanocomposite materials, the surface properties can also be influenced by the chemical composition, wettability and/or changing surface topography of the LDPE nanocomposite material. In addition, the appropriate choice of nanofiller types and their homogeneous distribution in the LDPE matrix makes it possible to influence the structural, antibacterial, frictional and/or mechanical properties.

With regard to tribological properties, the LDPE nanomaterials for medical devices (often moulded and extruded devices) achieve a lower coefficient of friction (COF), with values between 0.3 and 0.6, which implies better part lubricity and less overall friction between components and contact surfaces. Based on this aspect, LDPE (nano)material-based medical devices are used mainly in two sectors, where (1) they are not commonly exposed to or immersed in liquids, such as handle triggers or buttons, and where (2) they require wet lubrication, in contact with blood or fluids, predominantly in medical catheters [8,9].

The properties of LDPE nanocomposite materials such as their lightweightness, chemical inertness [10] and impermeability make them ideal for catheter tubing [1] as well as for packaging materials, such as coil hoops or sterile blister packs for drug packaging. They are flexible and can withstand repeated flexing over long periods [11]. Unfortunately, the wider use of LDPE nanomaterials in medicine is limited by the nanofiller types, which cause a reduction in hardness and scratch and/or wear resistance [9,11].

Nanofillers that are used for the modification of the LDPE (nano)materials have an impact on many of their properties; they have the basic advantage of a higher surface-to-volume ratio and higher surface area, creating better adhesion between the LDPE polymer matrix and nanofillers. The effective dispersion of nanostructured layered silicates with high aspect ratios may improve the mechanical, thermal and barrier properties of polymers, even at very low concentration. Since LDPE has a hydrophobic character and would not normally bind tightly to polar compounds, natural inorganic compounds such as clay minerals are modified by organic reactive groups or compounds. Typical examples, mainly based on montmorillonite, possess thermal stability, good mechanical properties (including elongation at break) and microhardness [12,13,14]; according to [13], nanoclays containing erucamide act as agents to create compatibility between the polar molecules of erucamide and the non-polar LDPE, which minimizes the migration of organic molecules and makes the friction coefficient more uniform and stable on the surface. A very low concentration (max. 1.5%) of the silica nanoparticles, with their high adsorptive capacity, smooth mesoporous surface and large pore volume, largely prevents the formation of their agglomerates and leads to a homogenous distribution in the LDPE matrix. This creates better compactness, strengthening the intermolecular forces between the silica nanoparticles and LDPE matrices, and leads to enhancing the tensile strength, elongation at break, Young’s modulus and optical properties [15]. The organic compound chitosan enhances the antimicrobial properties against E. coli and shows good oxygen permeability properties [16]. Chitosan incorporated in an LDPE matrix improves the barrier properties of the LDPE and confers antimicrobial characteristics due to the antimicrobial action between the positively charged chitosan molecules and negatively charged microbial cell membranes, which makes it a very promising packaging material. The graphene, due to its high elastic modulus, is easily released from the LDPE matrices and creates a contact zone on the LDPE surface, where it has a function as a solid lubricant. The graphene nanostructures prevent direct contact with other surfaces and thereby reduce the coefficient of friction (COF) and increase the wear resistance, conferring much better friction and wear resistance compared with virgin LDPE material [17]. The carbon nanotubes in the LDPE matrix tend to remain as agglomerates and not be broken into tubular nanoparticles. For this reason, various dispersion media are used for good compatibility between the CNTs and LDPE, which also provides an absence of interfacial gaps. Enhanced interfacial interactions are responsible for the better stress transfer and enhanced interfacial adhesions, which create better mechanical properties such as higher Young’s modulus values and/or electrical properties. The enhanced crystallinity and thermal stability of the LDPE/carbon nanotube nanocomposite materials are attributed to the enhanced interaction between the nanotubes and LDPE, where nanotubes may act as nucleation sites. It is believed that the crystalline regions enhance the stress transfer and, hence, the overall composite mechanical properties [18,19]. Zinc oxide nanoparticles, as photocatalytic nanomaterials, affect the LDPE nanocomposite surface by the formation of low-molecular-weight compounds such as hydroperoxides, peroxides, and carbonyl and unsaturated groups, resulting in increased brittleness along with wrinkles, cracks and cavities on the LDPE surface. These surface defects positively contribute to the antimicrobial nature of the LDPE/zinc oxide nanocomposite material, but also negatively contribute to their mechanical characteristics [20].

In this work, attention is focused on modification using hybrid nanofillers (inorganic/organic nanofillers) since their morphological and antimicrobial properties are known [21]. Zinc oxide (ZnO) and chlorhexidine (CH) nanoparticles are known for their antimicrobial behaviour; vermiculite (as a natural clay mineral/material) is an inert carrier for both components (ZnO and CH). It can be expected that their complete incorporation into the LDPE matrix ensures the antimicrobial nature of the LDPE nanocomposite materials and also increases the tribo-mechanical properties and thus allows wider use for medical applications. The combination of the individual nanofiller components (vermiculite, zinc oxide and chlorhexidine) vs. the LDPE matrices has not yet been published anywhere and is original.

The main aims of the present work were to develop a new antimicrobial LDPE nanocomposite material with incorporated hybrid nanofillers based on ZnO, vermiculite and chlorhexidine; optimize the nanofiller concentration with the respect to the antimicrobial activity of the LDPE nanocomposites against *Staphylococcus aureus* and *Pseudomonas aeruginosa*; and understand the correlation between the microstructure, surface topography and tribo-mechanical properties. The condition of tribo-mechanical measurements was chosen to simulate short-term (frictional) loading and to simulate the behaviour of nanomaterials at the most exposed time of their application.

## 2. Materials and Methods

### 2.1. Preparation of ZnO–Vermiculite–Chlorhexidine Nanofillers

The ZnO–vermiculite–chlorhexidine nanocomposite samples (nanofillers) were prepared by the sonochemical method, followed by a heat treatment and intercalation method realized by ultrasound. Ball-milled natural Mg-vermiculite from Brazil (supplied by Grena Co., Veselí nad Lužnicí, Czech Republic) with a mean particle diameter of 13.48 µm (*d*_43_) was used as a starting material for the nanocomposite sample preparation (sample named V).

The zinc oxide/vermiculite (ZnO/V) nanocomposite was prepared by the sonochemical method, followed by a heat treatment. Briefly, 7 g of vermiculite, 21 g of dihydrate zinc acetate ((CH_3_COO)_2_Zn·2H_2_O) and 21 g of anhydrous Na_2_CO_3_ (all from Sigma Aldrich, Czech Republic) were gradually added to the 200 mL of distilled water. A titanium sonotrode (UP100H from Hielscher, Teltow, Germany) was placed in the suspension, and it was sonicated for 15 min, at a 50% amplitude throughout the cycle. Subsequently, the sample was washed twice with distilled water and centrifuged until the chlorides disappeared. The solid nanocomposite material was dried and homogenized. The homogenized sample was calcined at 350 °C for 1.5 h.

The ZnO/V_CH nanocomposite was prepared by the intercalation of chlorhexidine dihydrochloride (CH) by ultrasound action. Briefly, 2 g of the ZnO/V nanocomposite was mixed in 50 mL of demineralized water for 3 min and then added to 50 mL of ethanol solution, in which 2 g of CH had previously been dissolved. The ultrasonic titanium sonotrode was placed in the dispersion in a beaker, and the mixture was subjected to ultrasound treatment for 15 min. The final dispersion was separated from the water/ethanol solution by centrifugation and drying at 75 °C for 24 h.

### 2.2. Preparation of the LDPE Nanocomposite Plates

The polyethylene matrix was prepared from the powdered and granulated mixture of the industrial low-density polyethylene without additives (LDPE, Bralen RB 2-62L, Slovnaft Co., Bratislava, Slovak Republic). The LDPE nanocomposite plates were prepared from the mixtures containing 40 g of LDPE granulates and 0.15 g (1 wt %), 0.45 g (3 wt %) and 0.75 g (5 wt %) of ZnO/V and/or ZnO/V_CH nanofillers. Each nanocomposite was prepared by a melt-compounded procedure in the mini compounder HAAKE MiniLab; the mixtures were blended at 150 °C for 15 min at a 50 rpm velocity. The final mass was pressed at 160 °C into 1 mm-thick plates of size 100 × 100 mm. The nanocomposite plates are denoted as LDPE_1% ZnO/V, LDPE_3% ZnO/V and LDPE_5% ZnO/V (and, respectively, LDPE_1% ZnO/V_CH, LDPE_3% ZnO/V_CH and LDPE_5% ZnO/V_CH).

### 2.3. Characterization Methods

The chemical composition of the V, ZnO/V and ZnO/V_CH nanofillers was obtained from elemental analysis by X-ray fluorescence spectroscopy (using the SPECTRO XEPOS energy-dispersive X-ray fluorescence spectrometer (Helmut Fischer GmbH, Kleve, Germany). The organic carbon content in the ZnO/V_CH nanofiller was determined using the phase carbon analyser RC612 (LECO, MI, USA). The defined weight of the ZnO/V_CH nanofiller was found by burning it in an oxygen atmosphere with a temperature range of 100 to 1000 °C. The carbon was detected in the IR cells in the form of CO_2_.

The morphology of the nanocomposite samples was investigated using a scanning transmission electron microscope (STEM, JEOL JSM-7610F Plus, Tokyo, Japan). The samples were coated with a gold/palladium film in order to avoid problems with electrical charging. SEM images were obtained using a scattered electron detector (SE, LEIUSA).

The particle sizes of the V, ZnO/V and ZnO/V_CH nanofillers were determined with the HORIBA laser diffraction particle size analyser (LA-950 instrument, Kyoto, Japan) with a two-short-wavelength blue- and red-light source in conjunction with forward and backscatter detection. The particle size analyses were conducted with the refractive indices 1.54 (for vermiculite) and 1.33 (for water).

The zeta potentials (*ξ*-potentials) of the V, ZnO/V and ZnO/V_CH nanofillers were measured using a nanoparticle analyser (HORIBA Nanopartica SZ-100, Kyoto, Japan) equipped with a microprocessor unit to directly calculate the *ξ*—potential. Of each sample, 0.5 g was mechanically mixed with 50 mL of distilled water. Then, 1 mL of the suspension was introduced into a disposable zeta potential cell. The *ξ*—potential was measured at natural pH. Each data point is an average of approximately 8 measurements. All the measurements were performed at ambient temperature (24.9 °C), conductivity (0.241 mS.cm^−1^) and suspension viscosity (0.90 mPa.s) and a constant electrode voltage (3.4 V).

The specific surface area (SSA) was measured in a nitrogen atmosphere by means of the Thermo Scientific Surfer. Prior to the measurements, the samples were degassed under vacuum (10^−6^ bar) at 120 °C for 24 h. The SSA was calculated using the BET (Brunauer–Emmett–Teller) equation by assuming the area of the nitrogen molecule to be 0.1620 m^2^.

The X-ray powder diffraction (XRD) analysis of all the experimental samples was performed using the diffractometer RIGAKU Ultima IV (CuKα radiation, NiKβ filter, Bragg–Brentano arrangement, scintillation detector, Tokyo, Japan). The samples, in a standard holder, were measured in an ambient atmosphere, with the operating conditions of 40 kV and 40 mA. The samples were measured in the 2θ range of 2–70° with a scanning rate of 3.8°/min). The phase analysis was evaluated using the database PDF-2 Release 2011. The figures of the XRD patterns were drawn using the Origin8Pro software.

The surface topography of the LDPE and LDPE nanocomposite plates and arrangements of the V, ZnO/V and ZnO/V_CH nanofillers in the LDPE matrix were investigated using a scanning electron microscope (SEM, QUANTA 450 FEG, FEI, Netherlands). The samples were coated with a gold/palladium film in order to avoid problems with electrical charging. The SEM images were obtained in a low vacuum using a secondary electron detector (SE). A plate area of 2.5 × 2.5 mm was characterised.

The surface topography and roughness of the surfaces of the LDPE nanocomposite plates were studied using a SolverNEXT (NT-MDT) atomic force microscope (AFM, Zelenograd, Russia). Non-contact mode with an 8 μm z-linearized dry scanner and a silicon probe 1650-00 was employed for AFM scanning. The images and roughness dates were evaluated using the Gwyddion 2.28 software.

The IR spectra of the powder samples were measured by the potassium bromide pellet technique. Exactly 1.0 mg of sample was ground with 200 mg of dried potassium bromide. This mixture was used to prepare the potassium bromide pellets. The IR spectra were collected using an FT-IR spectrometer, Nicolet iS50 (ThermoScientific, Waltham, MA, USA), with a DTGS detector. The measurement parameters were the following: spectral region, 4000–400 cm^−1^; spectral resolution, 4 cm^−1^; 64 scans; and Happ–Genzel apodization. The IR spectra of the LDPE nanocomposite samples were measured by the ATR (attenuated total reflectance, USA) technique. The samples were laid and pressed with a pressure device on a single-reflection diamond ATR crystal. The IR spectra were collected using an FT-IR spectrometer, Nicolet iS50 (ThermoScientific, USA), with a DTGS detector on a Smart Orbit ATR accessory. The measurement parameters were as follows: spectral region, 4000–400 cm^−1^; spectral resolution, 4 cm^−1^; 64 scans; and Happ–Genzel apodization.

### 2.4. Antimicrobial Activity

The antimicrobial activity (AC) of all the powder samples was tested against the Gram-positive strain *Staphylococcus aureus (S. aureus*, CCM 3953) and the Gram-negative strain *Pseudomonas aeruginosa (P. aeruginosa,* CCM 1960), provided by the Czech Collection of Microorganisms (CCM). The results were determined using a microbial fingerprints technique, which enabled determining the minimum inhibitory concentration (MIC) that completely inhibited bacterial growth in accordance with the lowest concentration. This method of direct fingerprints assumes that the microbes under the same conditions gradually die.

The AC of the LDPE nanocomposite plates was tested as follows. The colonies of bacterial culture were incubated for 20 h on blood agar and then were transferred into glucose broth. After 2 h of incubation, the colonies were diluted with physiological solution to reach a final dilution of 1.0 × 10^5^ CFU·mL^−1^, where the CFU (colony forming units) are the number of viable bacterial units in the colony. Each sample plate was cut into three square plates (25 cm^2^). The bacterial suspension (volume of 25 µL) on the individual LDPE plate surface was left to dry in a laminar box at 21 °C for 24 h. Then, the dried bacterial suspension was gradually stamped using the microbial fingerprints technique on the three disks with blood agar. The first three fingerprints of dry suspensions were made after 24, 48, 72 and 96 h, respectively. All the experiments were triplicated, and average data were produced.

### 2.5. Tribo-Mechanical Properties

The microhardness in different places of the LDPE nanocomposite plates was evaluated using a Vickers microhardness tester (Micro Combi Tester, MHT3, Anton Paar, Switzerland). The indentor was a square-shaped diamond pyramid with a top angle of 136°. In this study, the microhardness tests were performed under an applied load of 500 mN, and the loading time was 90 s. The result values are the average results of 10 imprints. The instrumentation hardness testing was performed according to CSN EN ISO 14577.

The LDPE nanocomposite samples were tested with the mechanical tester UMT Tribolab (Bruker Corporation,) and optical profilometer Contour GTX (Bruker Corporation) by the ball-on-flat method. As the balls, steel balls with a diameter of 0.20 mm and microhardness of 60 HRC were used. The stroke length of the reciprocating movement was set as 10 mm. Then, two sets of measurements were carried out. The first was with a 2 N loading force and speed of 5 Hz for 5 min, which meant 3000 passes over the sample surface; the test was named F2N_v5. The second test was with a 1 N loading force and speed of 1 Hz for 5 min, which meant 480 passes over the sample surface. This test was named F1N_v1. The wear track depth was evaluated using a 5× objective in VSI mode. Tilting of the measurement data was performed by plane fitting, and the measured points were evaluated using the Legacy method.

## 3. Results and Discussion

### 3.1. ZnO–Vermiculite–Chlorhexidine Nanofiller Characterization

**Figure 1:**
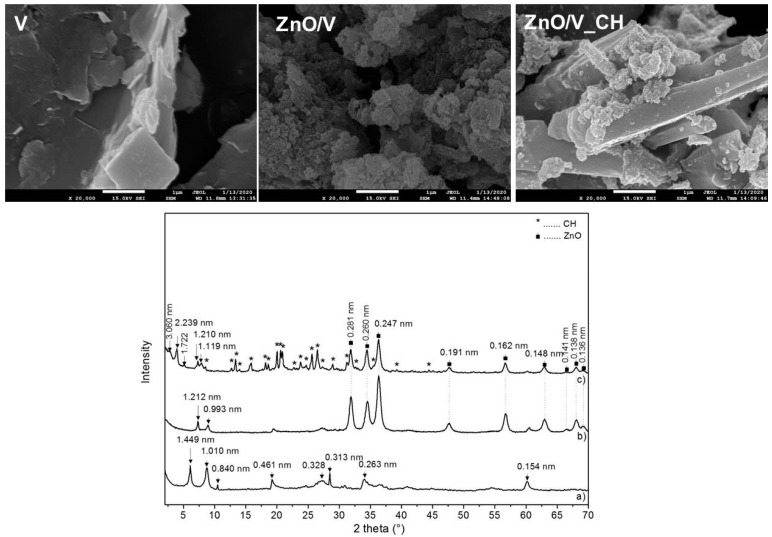
SEM images and XRD patterns of the nanofiller samples: (a) V, (b) ZnO/V and (c) ZnO/V_CH.

Natural Mg–vermiculite (V), determined to have narrow monomodal particle size distributions, with a mean diameter *d*_43_ = 13.48 µm, specific surface area (SSA) of 32.02 m^2^·g^−1^ and ξ-potential value of −60.0 mV, was used as the starting material. The V particles had irregular shape with a smooth surface and round and deformed edges (Figure 1). The structural formula of the V sample was calculated on the basis of elemental chemical analysis: (Si_6.32_Al_1.58_Ti_0.1_) (Mg_4.75_Ca_0.34_Fe_0.91_) O_20_ (OH)_4_ (Ca_0.04_ K_0.38_).

The ZnO/V nanocomposite particles exhibited homogenous ZnO nanoparticle growth on the vermiculite particle’s edges and surfaces (Figure 1). The agglomeration rate of the ZnO/V nanocomposite particles was confirmed with a value of the surface charge, ξ-potential value, of −20.6 mV. On the basis of the particle size analyses, it was found that the sonochemical process and heat treatment caused a reduction in the V particles with diameter d_43_ = 9.07 µm and with SSA = 20.23 m^2^·g^−1^. From the elemental chemical analysis, the presence of 21.09 wt% of the ZnO nanoparticles in the ZnO/V particles was confirmed.The ZnO/V_CH nanocomposite particles were formed by the vermiculite particles with a very smooth surface and sharp edges, at the ends of which ZnO nanoparticles were preserved (Figure 1). The ZnO/V_CH particles exhibited narrow monomodal particle size distributions, with mean diameter d_43_ = 10.97 µm, an SSA of 24.30 m^2^·g^−1^ and a ξ-potential value of +23.5 mV. The ultrasound intercalation of CH led to a decrease in ZnO composition from 21.09 wt% (ZnO/V) to 16.33 wt% (ZnO/V_CH), which was supported by the organic carbon content having the value of 27.47 wt%.

X-ray diffraction (XRD) analysis confirmed the successful preparation of the ZnO/V and ZnO/V_CH nanocomposite samples. The XRD pattern of V (Figure 1) shows that the vermiculite phase (ICDD PDF card no. 01-074-1732) at *2θ* = 6.09°, 19.21°, 27.16°, 33.19° and 60.11° corresponds to *d*-values of 1.449, 0.461, 0.328, 0.263 and 0.154 nm, and an admixture phase of tremolite (ICDD PDF card no. 00-013-0437) at *2θ* = 10.52° and 28.46° corresponds to *d* = 0.840 and 0.313 nm. The reflection at *2θ* = 8.75° with *d* = 1.010 nm belongs to the dehydrated phase of the V structure after calcination at 350 °C. The XRD pattern of ZnO/V shows a shift of V’s basal reflections to 7.29° *2θ,* with *d* = 1.212 nm. This was caused by the cationic exchange of interlayer materials for ZnO during nanocomposite preparation. The reflections of the hexagonal wurtzite structure of ZnO (PDF card no. 01-079-2205) at *2θ* = 31.87°, 34.46°, 36.35°, 47.58°, 56.66°, 62.90°, 66.38°, 68.05° and 69.25° correspond to *d*-values of 0.281, 0.260, 0.247, 0.191, 0.162, 0.148, 0.138 and 0.136 nm, confirming the ZnO nanoparticles on V’s surface.

The XRD pattern of ZnO/V_CH shows, in addition to the reflections of V and ZnO, new reflections at *2θ* = 2.88°, 3.94° and 5.12°, with *d*-values of 3.060, 2.239 and 1.722 nm, respectively, and 1.119 nm as a result of CH intercalation into the V interlayer [21,22]. Reflections of non-intercalated CH on the V surface are also observed.

The ZnO crystallite sizes calculated according to Scherrer’s equation [23] based on (100) reflection (2*θ* = 31.78°) are 14.33 nm for ZnO/V and 20.31 nm for ZnO/V_CH.

**Figure 2:**
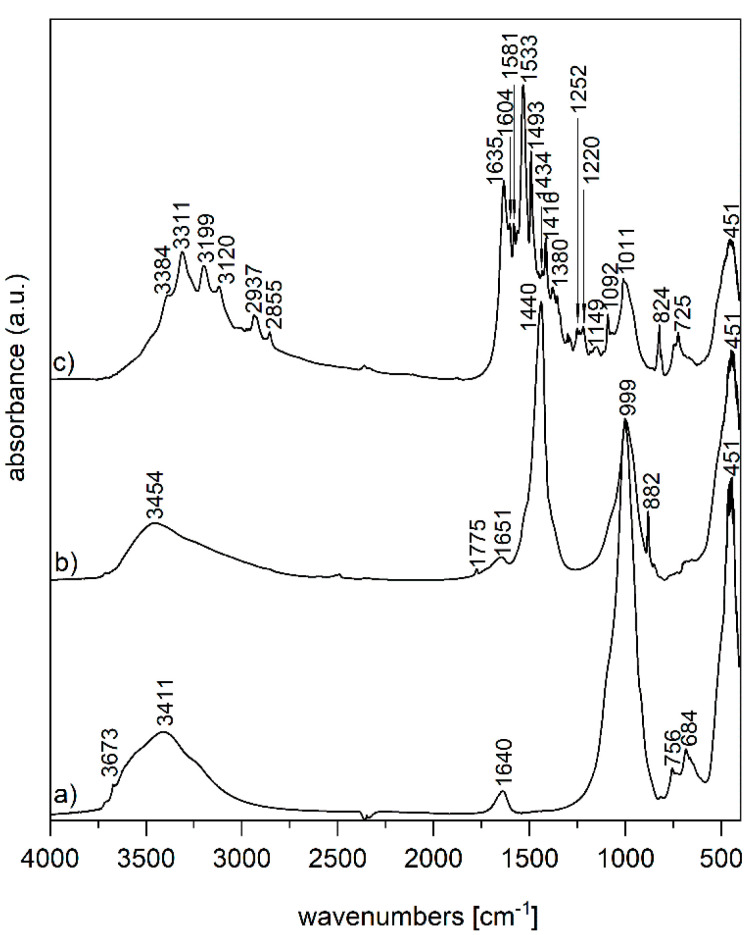
FTIR spectra of the nanofiller samples: (a) V, (b) ZnO/V and (c) ZnO/V_CH.

The FTIR spectrum of the initial V (Figure 2a) shows a band at 3673 cm^−1^ in the O–H stretching region attributed to the Mg_3_OH unit, together with absorption at 684 cm^−1^ belonging to the O–H bending vibration; these bands suggest a trioctahedral character for V [24]. The absorption observed at 3411 cm^−1^ corresponds to O–H stretching vibrations of absorbed water, and those at 1640 cm^−1^, to the O–H bending vibration of absorbed water. The intense band at 999 cm^−1^ was assigned to Si–O stretching vibrations, together with the Si–O bending vibration at 451 cm^−1^ [24]. In the FTIR spectrum of ZnO/V (Figure 2b), the band at 1440 cm^−1^ is the overlap of both peaks for C-H and C=O bonds originating from acetate; a further stretching mode of vibration of the C=O bond is observed at 882 cm^−1^ [25]. The main Zn–O stretching band is overlapped by the Si–O bending vibration of V. The FTIR spectrum of ZnO/V_CH (Figure 2c) shows bands at 3311, 3199 and 3120 cm^−1^ corresponding to NH stretching vibrations of secondary amine and imine functional groups of CH; further bands at 2937 and 2855 cm^−1^ belong to asymmetric and symmetric C–H stretching bands of CH. The C–N stretching vibration of an imine group appears at 1635 cm^−1^. The bands occurring in the 1580–1490 cm^−^^1^ interval are due to a N–H bending vibration of secondary amine and imine groups. The absorption at 1416 cm^−^^1^ belongs to the C–C stretching vibrations of an aromatic ring. Finally, the bands at 824 and 725 cm^−^^1^ belong to the C–H out-of-plane deformation vibrations of 1,4-disubstituted aromatic rings and C-C rocking vibration of methylene groups, respectively [26,27].

### 3.2. LDPE Nanocomposite Plate Characterization

**Figure 3:**
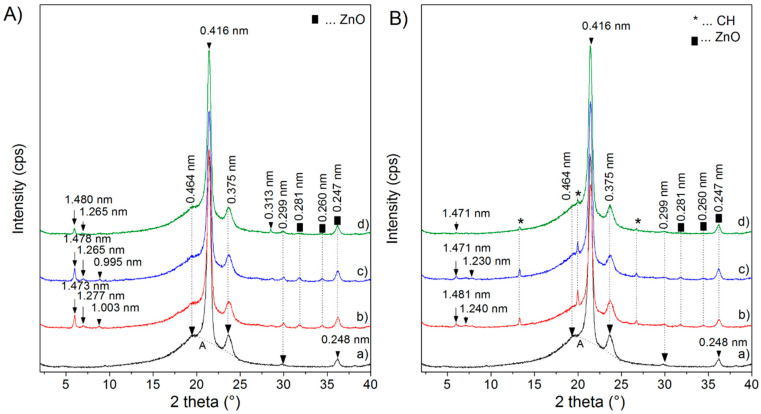
XRD patterns of the low-density polyethylene (LDPE) nanocomposite plates. (**A**): (a) LDPE, (b) LDPE_5% ZnO/V, (c) LDPE_3% ZnO/V, (d) LDPE_1% ZnO/V; (**B**): (a) LDPE, (b) LDPE_5% ZnO/V_CH, (c) LDPE_3% ZnO/V_CH, (d) LDPE_1% ZnO/V_CH.

The XRD pattern of the LDPE nanocomposite plates (Figure 3) shows a semicrystalline structure for LDPE with the reflections of the crystalline region at 2*θ* = 21.35°, 23.71°, 29.83° and 36.20° corresponding to *d* = 0.416, *d* = 0.375, *d* = 0.299 and *d* = 0.248 nm and also the amorphous region (A) between 15 and 25° 2*θ* [28]. The XRD pattern of the samples with nanofillers does not show significant changes in the LDPE reflections.

The LDPE nanocomposite samples with the ZnO/V nanofiller show V reflections with *d*-values of 1.277–1.265 and 1.473–1.481 nm, which means a partially rehydrated V structure. ZnO reflections with *d*-values of 0.281, 0.260 and 0.248 nm are observed (Figure 3A).

The LDPE nanocomposite samples with the ZnO/V_CH nanofiller show very low intense V reflections with *d*-values of 1.230–1.240 and 1.471–1.481 nm corresponding to the rehydrated V structure. However, the reflections 3.060, 2.239 and 1.722 nm disappeared in the LDPE nanocomposites with ZnO/V_CH nanofiller, which could mean a partially exfoliated V structure in the LDPE and the formation of a nanocomposite structure. ZnO and CH reflections are observed in the XRD patterns (Figure 3B).

**Figure 4:**
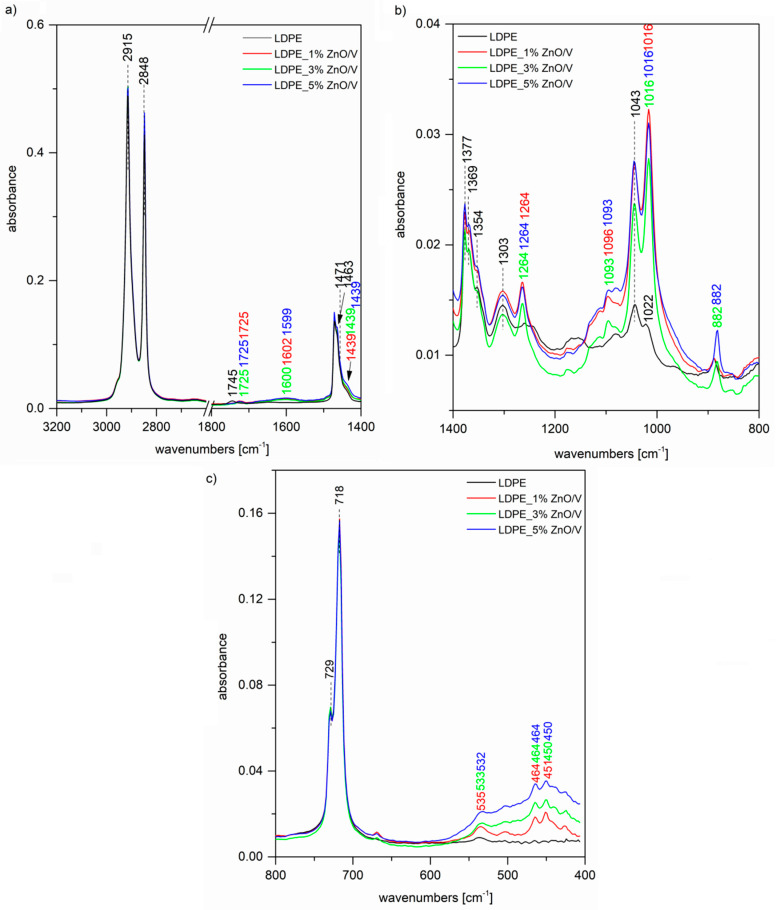
FTIR spectra of the original LDPE and LDPE_ZnO/V nanocomposite plates at regions (**a**) 3200–1400 cm^−1^, (**b**) 1400–800 cm^−1^ and (**c**) 800–400 cm^−1^.

**Figure 5:**
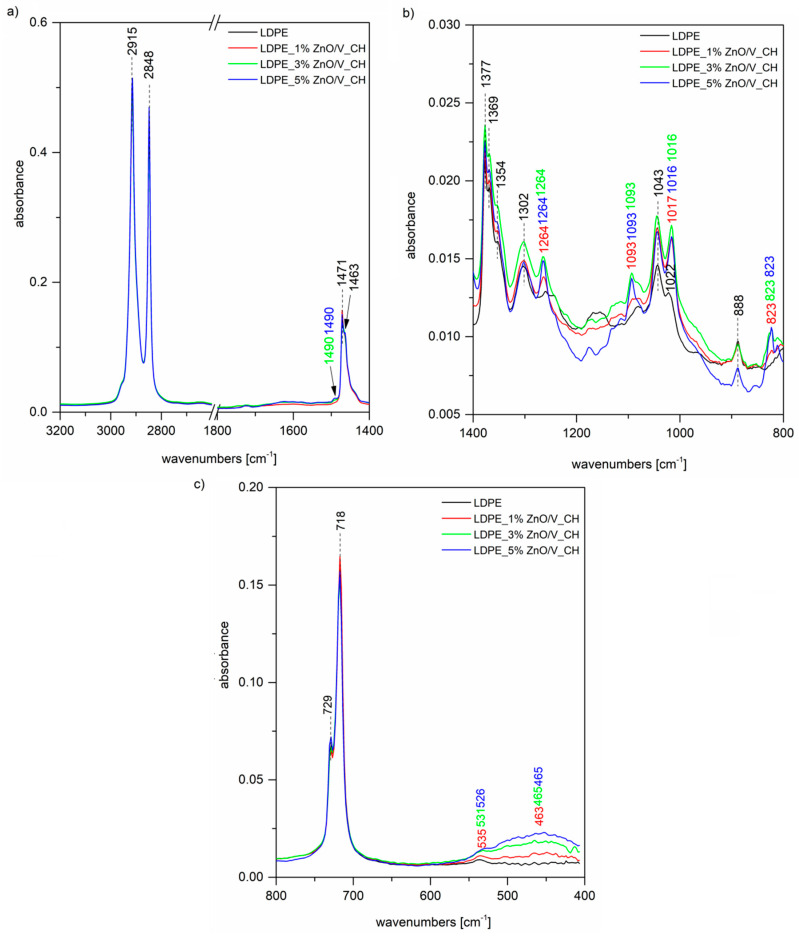
FTIR spectra of the original LDPE and LDPE_ZnO/V_CH nanocomposite plates at regions (**a**) 3200–1400 cm^−1^, (**b**) 1400–800 cm^−1^ and (**c**) 800–400 cm^−1^.

The FTIR spectrum of the original LDPE (Figure 4a) shows two intense peaks at 2915 and 2848 cm^−1^ assigned to C–H asymmetric and symmetric stretching vibrations of methylene groups CH_2_. A further two bands of the doublet 1473 and 1463 cm^−1^ belong to the bending deformation of methylene CH_2_ groups in crystalline and amorphous domains, respectively [26,27,29,30]. The band at 1377 cm^−1^ (Figure 4b) is assigned to the CH_3_ symmetric deformation vibration, the peaks at 1369 and 1354 cm^−1^ are both assigned to the wagging deformation vibration of CH_2_, and 1303 cm^−1^ is assigned to twisting deformation vibration. The weak peak at 1745 cm^−1^ (Figure 4a) together with that at 1043 cm^−1^ (Figure 4b) is correlated with the presence of the carbonyl C=O absorption and CH_2_–O vibration of the ester group, which is probably created by the natural aging of LDPE [30]. Finally, the peaks of the doublet 729 and 718 cm^−1^ (Figure 4c) are assigned to CH_2_ rocking deformation vibrations in crystalline and amorphous domains. In the case of LDPE_ZnO/V nanocomposites, we can observe in the FTIR spectra characteristic bands for the ZnO/V nanofiller. The shoulder on the intensive peak at 1471 cm^−1^ (Figure 4a) around 1439 cm^−1^ is the overlap of both peaks for C–H and C=O bonds originating from zinc acetate; a further stretching mode of vibration of a C=O bond is observed at 882 cm^−1^ (Figure 4b) for 3 and 5 wt% of the ZnO/V nanofiller. The bands around 535 and 464 cm^−1^ (Figure 4c) correspond to Zn–O stretching bonds. Although we did not observe these bands in the spectrum of the ZnO/V nanofiller itself (Figure 5) due to overlapping by more intense Si–O bending vibrations, after the incorporation into the LDPE matrix, it is possible that some changes in the nanofiller structural arrangement occurred and these bands are observable.

Figure 5 shows, as well as the previous Figure 4 spectrum of the original LDPE, further spectra of the LDPE_ZnO/V_CH nanocomposites. In this case, besides the LDPE characteristic bands, we can again observe vibrations of the ZnO/V_CH nanofiller. Especially, some vibrations of very dominant CH from the nanofiller are shown in the LDPE nanocomposites’ spectra at 1490 cm^−1^ (Figure 5a), attributed to the N–H bending vibration of secondary amine or imine groups, and 823 cm^−1^ (Figure 5c), which belongs to the C–H out-of-plane deformation vibrations of 1,4-disubstituted aromatic rings of CH.

**Figure 6:**
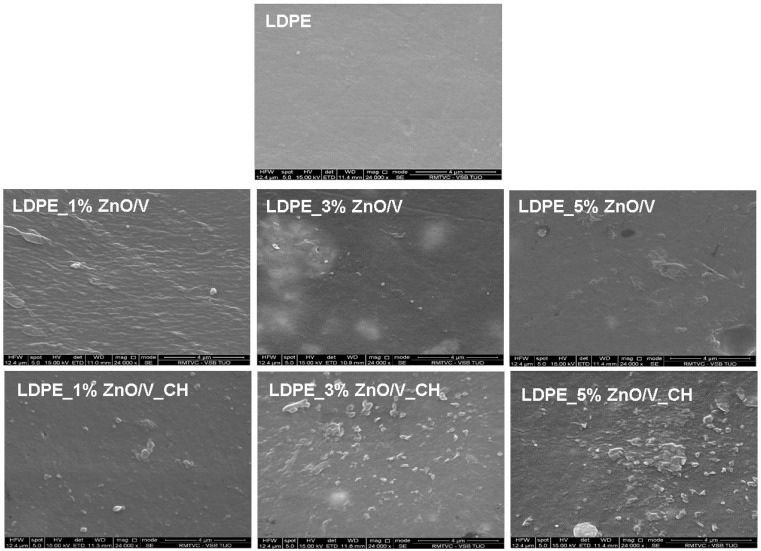
SEM images of the natural LDPE surface and LDPE_1% ZnO/V, LDPE_3% ZnO/V, LDPE_5% ZnO/V, LDPE_1% ZnO/V_CH, LDPE_3% ZnO/V_CH and LDPE_5% ZnO/V_CH nanocomposite surfaces.

The surface topology of the LDPE and LDPE nanocomposite plates was investigated using scanning electron microscope (SEM) and atomic force microscope (AFM) measurements. Figure 6 shows the cross-sectional SEM images representing the arrangement of the visible ZnO/V and ZnO/V_CH particles on the LDPE nanocomposite plates.

The original LDPE nanocomposite plates show a smooth surface with sporadically occurring holes and grooves as a result of the sample handling.

**Figure 7:**
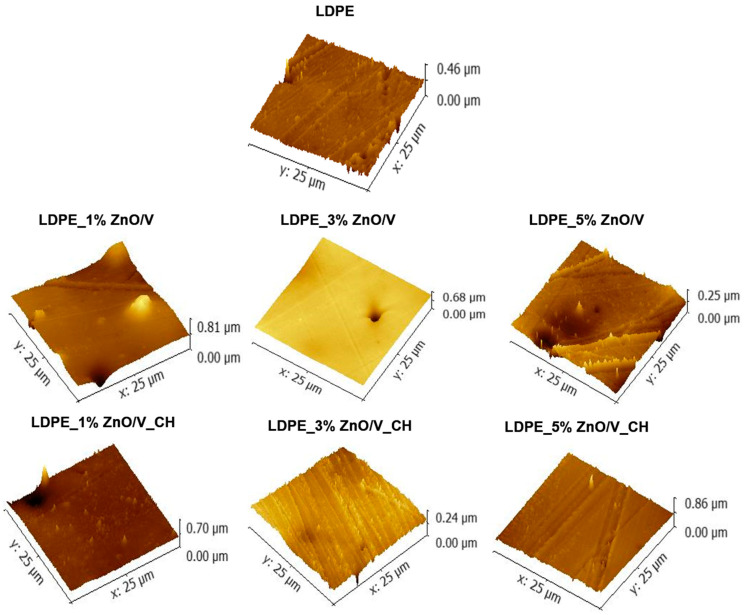
3D AFM images of the natural LDPE surface and LDPE_1% ZnO/V, LDPE_3% ZnO/V, LDPE_5% ZnO/V, LDPE_1% ZnO/V_CH, LDPE_3% ZnO/V_CH and LDPE_5% ZnO/V_CH nanocomposite surfaces.

From the SEM images (Figure 6) are evident the better incorporation and distribution of the ZnO/V nanofillers in the LDPE matrices’ volume than those of the ZnO/V_CH nanofillers. The LDPE_3% ZnO/V nanocomposite plate shows complete incorporation and the most homogeneous distribution of the ZnO/V nanofiller in the LDPE matrix. The ZnO/V nanofiller particles are uniformly distributed, and there are some areas of variable size and shape. Cavities smaller than 0.5 µm (also visible in the AFM image, Figure 7) are visible at the places of the agglomerates. The LDPE_3% ZnO/V nanocomposite plate was an optimized sample in terms of technological preparation. The 5% of ZnO/V nanofillers were also uniformly distributed, but they created agglomerates in the LDPE plate. The ZnO/V nanofillers were horizontally oriented, and it is evident that the nanofiller presence on the surface of the LDPE matrix (part of it was sticking out from the LDPE surface) contributed to the formation of the surface cavities and holes around them. The surface of the LDPE_1% ZnO/V nanocomposite plate was wavy, and sporadically, there were ZnO/V nanofiller particles with non-uniform shapes and sizes. At the same time, very small, spherical nanoparticles were visible on the surfaces of the LDPE_1% ZnO/V and LDPE_3% ZnO/V nanocomposite plates as a result of the agglomeration of nanoparticles from the conductive layer sputtering process during the surface preparation for SEM analysis.

From the SEM images (Figure 6) is evident the increased size of the weight fraction of the ZnO/V_CH nanofiller in the LDPE plates, where the ZnO/V_CH particles stuck out of the LDPE surface. In the case of the LDPE_1% ZnO/V_CH and LDPE_3% ZnO/V_CH nanocomposite plates, mainly ZnO/V_CH particles with an average size of 2 µm in the form of thin plates with sharp edges were anchored on the surface. The LDPE_5% ZnO/V_CH nanocomposite plate was represented by ZnO/V_CH particles with different particle size ranging from 0.5 to 2 µm, whereas particles bigger than 2 µm were partially embedded in the LDPE matrix and on the surface formed a sharp protrusion. On the LDPE_1% ZnO/V_CH and LDPE_3% ZnO/V_CH nanocomposite plates, surface defects were not observed; only in the case of the LDPE_5% ZnO/V_CH nanocomposite plate were short cracks (no longer that 2 µm) occasionally visible outside the area of the occurrence of the ZnO/V_CH nanofillers.

It can be stated that the predominant larger fractions (5–13 µm) of the ZnO/V and ZnO/V_CH nanofillers were incorporated inside the LDPE matrix, while smaller fractions (less than 4 µm) were anchored on the surface of the LDPE matrices, depending on the concentration amount.

The topology changes of the LDPE plates are shown in the 3D AFM images (Figure 7). Variations in morphology were calculated via the average roughness (Ra, measured by profile/linear analysis) and root mean square roughness (RMS) values (Table 1). All were obtained from the six measurements at different locations of the LDPE plates for each sample. The standard deviations are provided in parentheses.

From the 3D AFM images, it is evident that the LDPE nanocomposite plates reached maximum heights at around 0.25 µm (LDPE_5% ZnO/V and LDPE_3% ZnO/V_CH samples), 0.70 µm (LDPE_3% ZnO/V and LDPE_1% ZnO/V_CH samples) and 0.86 µm (LDPE_1% ZnO/V and LDPE_5% ZnO/V_CH samples). The highest measured values correspond with the perpendicular orientation (LDPE_1% ZnO/V sample) and the presence of agglomerates (LDPE_5% ZnO/V_CH samples) of the nanofillers on the LDPE surface, as has already been observed in the SEM images. The smoothest surfaces were characterized on the LDPE_1% ZnO/V, LDPE_3% ZnO/V and LDPE_5% ZnO/V_CH nanocomposite plates. In all the AFM images are evident maximal peaks that correspond to the presence of powdered gold from the covering of the LDPE plates. These could have only slightly affected the resulting numerical roughness values.

The roughnesses, with the lowest values of 21.7 nm (Ra) and 34.0 nm (RMS), were measured and evaluated for the original LDPE plate (Table 1). The ZnO/V and ZnO/V_CH nanofillers caused an increase in the Ra roughness values in the range of values from 24.68 nm (for the LDPE_5% ZnO/V_CH sample) to 29.3 nm (for the LDPE_3% ZnO/V sample). As a remote value of the measurement can be considered the average roughness of 36.45 nm (Ra) for the LDPE_1% ZnO/V sample, which was caused by the perpendicular orientation of the ZnO/V nanofillers on the surface of the LDPE matrix, as can be seen from the AFM image (Figure 7). For this reason, the mean values of the root mean square roughness (RMS) can be considered more objective, because they were evaluated from the total scanned area of the LDPE surface. Additionally, these values increased (RMS) in the range from 34.1 nm (for LDPE_5% ZnO/V_CH) to 50.9 nm (for LDPE_3% ZnO/V), respectively, and 57.8 nm for LDPE_1% ZnO/V. It is evident that with an increasing mass percentage of the nanofillers (ZnO/V and ZnO/V_CH) in the LDPE matrix, the roughness values decreased.

### 3.3. Antimicrobial Activity of the PVAc Nanocomposite Plates

The antimicrobial activity of the prepared LDPE nanocomposites was studied using the microbial fingerprints technique. Briefly, 25 μL of microbial suspension with a density of 10^5^ CFU/mL was spread on 2.5 cm^2^ surfaces of the tested square plates of LDPE nanocomposites, which meant that the original density of the suspension on the square plates dropped by one logarithmic order. The pure LDPE was used as the control sample. The average numbers of fungal colony forming units (CFU) at various time intervals are shown in Table 2.

The ZnO/V and ZnO/V_CH nanofillers themselves exhibited high antibacterial activity against *S. aureus* and *P. aeruginosa* after 30 min, with a long-lasting effect persisting up to 5 days, with a dependence on the zinc oxide and chlorhexidine concentrations in the vermiculite structure [31]. By incorporating them into the LDPE matrix, the antibacterial activity was changed slightly.

In the case of action against *S. aureus*, we can observe slightly improving efficacy over time with the samples LDPE_3% ZnO/V and LDPE_5% ZnO/V. All the other samples had fluctuating efficacies, without time and concentration dependence. The bactericidal effect was not confirmed.

We obtained very good results with all the LDPE nanocomposites against *P. aeruginosa* after 72 h of action, when we noted a decrease in CFU up to four logarithmic orders, and after 96 h, the number of CFU even decreased nearly to zero values. Surprisingly, there was no difference between the samples with and without the presence of CH. These results correspond with the antibacterial activity published in [27].

### 3.4. Tribo-Mechanical Properties of the LDPE Nanocomposite Plates

**Figure 8:**
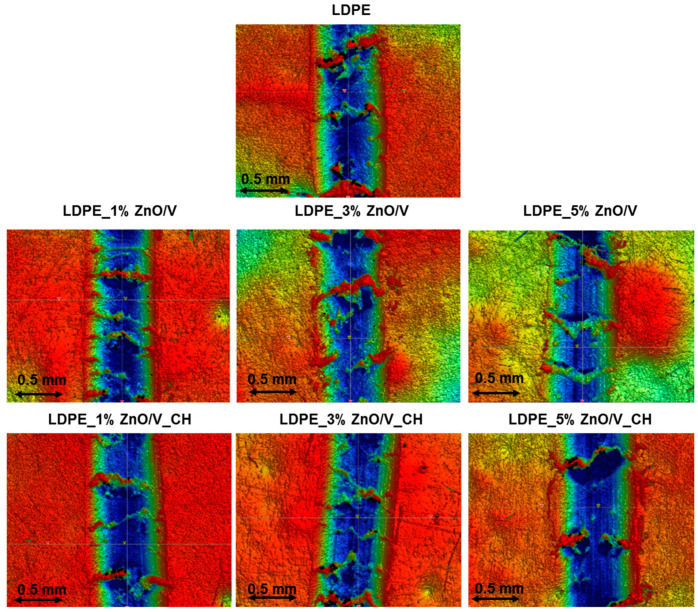
The optical profilometry images against a steel ball after F2N_v5 tests of the natural LDPE surface and LDPE_1% ZnO/V, LDPE_3% ZnO/V, LDPE_5% ZnO/V, LDPE_1% ZnO/V_CH, LDPE_3% ZnO/V_CH and LDPE_5% ZnO/V_CH nanocomposites.

The tribological behaviour of the LDPE samples was evaluated by static wear tests performed against steel balls using a ball-on-disc micro tribometer for two different conditions—a 2 N loading force and speed of 5 Hz (test named F2N_v5), and a 1 N loading force and speed of 1 Hz (test named F1N_v1). Both tests were realised for 5 min. The resulting friction coefficients (COF, average values measured during the test based on five repetitions of each test were evaluated, with the minimum standard deviations ±0.05) and abrasion depths (standard deviations ±0.3 µm) are summarized in Table 3. The representative profilometry images from F5N_v5 are shown in Figure 8.

During the first 10 s of the tribological measurements (considered as the run-in period), the COF values always increased to the maximum, for all the LDPE samples. The highest COF value, 0.675, was measured for LDPE_3% ZnO/V_CH, followed by 0.665 for LDPE_5% ZnO/V and LDPE_3% ZnO/V, 0.64 for LDPE_5% ZnO/V_CH and LDPE_1% ZnO/V_CH, and 0.590 for LDPE and LDPE_1% ZnO/V. Then, the COF values decreased gradually and after 150 s reached a steady value. They correspond with the theory published in [32], when after the run-in period, the contact surface of the tested balls became smoother, and prominent asperities of the surface were flattened or removed. The tribological plots (COF vs. time) are not listed here. The COF values measured at the end of the measuring time are shown in Table 3.

The COF values of the LDPE nanocomposites from both wear tests were in the range, in terms of average values, of 0.451 to 0.560, compared to the original 0.454 to 0.507 for the natural LDPE plate. The COF values of the ZnO/V and ZnO/V_CH nanofillers in the LDPE matrix slightly increased during the F1N_v1 test (to a maximum COF value of 0.560 for LDPE_1% ZnO/V_CH) compared to the F2N_v5 test. In both tests, with an increasing concentration of the ZnO/V_CH nanofiller in the LDPE nanocomposite, the COF value slightly decreased, while in the ZnO/V nanofiller, this trend was the opposite.

From the tribological values, it is evident that the friction coefficient (COF) is a factor that expresses the surface characteristics of the LDPE nanocomposites and is higher for rougher surfaces (COM vs. RMS values). This fact is also confirmed by the measured values of the abrasion depth after the tribological test, which indicate the abrasion resistance, the ability of a surface to resist being worn away, upon rubbing or friction on the LDPE surface defect created after contact with the steel ball.

The abrasion depths of the wear obtained for the natural LDPE were around 11 µm (measured only after the F2N_v5 test). The average wear depth of the tracks for the ZnO/V nanofiller slightly decreased from 9.7 to 9.3 µm. Bigger abrasion depths were obtained for the ZnO/V_CH nanofillers, ranging from 11.6 to 14.1 µm, the biggest abrasion depth of 14.1 µm being measured for the LDPE_3% ZnO/V_CH nanocomposite plate. These results indicate that the LDPE_ZnO/V nanocomposite plates exhibited better wear resistance. They are also in good agreement with the profilometry images (Figure 8), where are clearly evident structurally inhomogeneous areas with a lot of debris in the wear track. They are caused by the destruction of the samples relatively soft in comparison with the hard steel ball. This fact is also evident on the LDPE_ZnO/V_CH plates, where after the frictional tests, the material was displaced at the wear track interface. It is reflected on the images of the optical profilometer by a distinctive red colour in contrast to the blue colour of the wear track.

From the tribological results, it can be assumed that LDPE nanocomposites with ZnO/V nanofillers show better tribological properties due to higher COF (and RMS) values and lower values of abrasion depth, regardless of the degree of loading during the tribological test. The LDPE_ZnO/V nanocomposite plates better resisted frictional failure, the ZnO/V nanoparticles absorbing some of the slip, leaving less to migrate to the surface of the LDPE. On the contrary, the ZnO/V_CH nanofillers generated lower COF values and created deeper wear on the surface of the LDPE boards. This fact may be due to the presence of ZnO/V_CH nanofillers situated on the surface of the LDPE nanocomposite plates, where the predominantly layered structure of vermiculite particles and presence of the CH nanoparticles (organic chains) can act as lubricant agents and thus soften the surface of the LDPE plates.

The mechanical properties of the LDPE plates were determined using the indentation hardness (HIT) and Vickers microhardness (HV); the maximum achieved impression depth (hm) was measured, and the results are summarized in Table 4. For the purpose of providing reliable microhardness values corresponding to the total hardness of the nanocomposites, the imprints under each load should be larger in size than the dimensions of the dispersed ZnO/V and ZnO/V_CH nanofillers (around 10 µm). It is known that the mean diagonal length of the imprints varies in the range of 100–200 μm. The measured values of the maximum impression depth (hm) ranged from 31.8 to 34.8 µm. The highest value of 41.9 µm was measured for the LDPE_3% ZnO/V_CH nanocomposite plate. This means that the imprints were always greater than the dimensions of the dispersed nanofillers. It is evident that both nanofillers led to a slight increase in the HIT and HV values.

The HIT value increased from 20.9 MPa (for the original LDPE) to 27.2 MPa (for LDPE_1% ZnO/V_CH) and 27.2 MPa (for LDPE_1% ZnO/V_CH), and the HIT value decreased with increasing concentrations of both nanofillers. These values corresponded with the Vickers microhardness values, where the original 2.0 HV for LDPE increased to 2.6 HV for LDPE_1% ZnO/V and also LDPE_1% ZnO/V_CH.

Different microhardness values were measured for the PVDF_3% ZnO/V_CH, where the microhardness reached significantly lower values of 1.6 HV and 16.7 MPa (HIT value). This indicates significantly softer/plastic behaviour. This also corresponds to the maximum impression depth (hm = 41.9 MPa), which was significantly deeper than for the other PVDF nanocomposite samples.

## 4. Conclusions

The effects of two types of nanofillers (ZnO/V and ZnO/V_CH) on the structural, phase, topology, tribo-mechanical and antimicrobial changes in the LDPE nanocomposite plates were examined. Nanofillers with almost identical particle sizes (d_43,_ 9.07 and 10.97 µm) and specific surface areas (SSA, 20.23 and 24.30 m^2^·g^−1^) and different ξ-potential values −20.6 mV for ZnO/V and +23.5 mV for ZnO/V_CH nanofillers) were incorporated into the LDPE matrix in amounts of 1, 3 and 5 wt% via melt compounding and hot press methods.

XRD analysis confirmed the good interaction of both the nanofillers with the LDPE matrices. The detailed FTIR analysis revealed a new aspect of the interaction of OH ions (as a result of the incomplete reaction of the preparation of ZnO nanoparticles from zinc acetate) and the changes in the orientation of the CH molecule (linear chain vs. core) during the LDPE nanocomposite preparation processes. These aspects probably made a positive contribution to the final tribo-mechanical properties.

The SEM micrographs showed that both types of nanofillers were well integrated into the volume of the LDPE matrix, but the finest fractions of the ZnO/V_CH nanofillers (which did not exceed 2 micro) were anchored on the surface of the LDPE nanocomposite plates. The AFM measurements confirmed the smoothest surfaces of the LDPE_1% ZnO/V and LDPE_3% ZnO/V and an increase in the average roughness (RMS) to 50.9 nm for LDPE_3% ZnO/V, unlike the original 34.0 nm of the natural LDPE nanocomposite plate. Higher HV (~2.6) and HIT (~26 MPa) microhardness values were measured for both nanofillers than the natural LDPE nanocomposite plate (HV = 2.0, HIT = 20.9 MPa), even though with an increasing concentration of ZnO/V nanofiller, these values slightly decreased. The microhardness changes were also reflected in lower maximum impression depth values, which fell from the original 37 micrometres to an average of 32 micrometres. The microhardness changes were also reflected in the lower values of the maximum impression depth (hm), which decreased from the natural 37 µm to an average of 32 µm. The ZnO/V and ZnO/V_CH nanofillers, regardless of the concentration in the LDPE matrix, slightly increased the average values of the friction coefficient (COF). The abrasion depths of the wear indicated that the LDPE_ZnO/V nanocomposite plates exhibited better wear resistance than LDPE_ZnO/V_CH.

All the LDPE nanocomposite plates displayed antimicrobial activity against *S. aureus* and *P. aeruginosa*; more positive effects were proven with the ZnO/V_CH nanofiller than for ZnO/V.

The antimicrobial LDPE nanocomposite materials with hybrid ZnO–vermiculite–chlorhexidine nanofillers represent a group of new materials with improved antibacterial and tribo-mechanical properties, they are easy processable, and they should increase the acceptance of polymer products mainly for medical devices and/or the packaging industry.

## Figures and Tables

**Table 1:** The average values of the surface roughness (Ra, RMS) evaluated from the AFM measurements of the LDPE plate surfaces. Standard deviations are provided in parentheses.

Samples	Ra	RMS
	(nm)	(nm)
LDPE	21.8 (0.5)	34.0 (0.8)
LDPE_1% ZnO/V	36.5 (3.1)	57.8 (4.1)
LDPE_3% ZnO/V	29.3 (1.5)	50.9 (3.0)
LDPE_5% ZnO/V	26.9 (1.1)	37.8 (1.4)
LDPE_1% ZnO/V_CH	25.7 (0.6)	45.9 (1.6)
LDPE_3% ZnO/V_CH	24.9 (4.1)	41.9 (5.7)
LDPE_5% ZnO/V_CH	24.7 (0.5)	34.1 (0.6)

**Table 2:** Efficiency of individual antibacterial agents against *S. aureus* and *P. aeruginosa.*

Samples	*S. aureus*				*P. aeruginosa*			
	Exposition Time (h)							
	24	48	72	96	24	48	72	96
LDPE_1% ZnO/V	368	313	122	289	179	59	30	2
LDPE_3% ZnO/V	577	305	195	154	249	108	12	1
LDPE_5% ZnO/V	549	279	133	118	191	42	0	3
LDPE_1% ZnO/V_CH	267	122	100	430	238	93	19	4
LDPE_3% ZnO/V_CH	108	111	70	710	192	104	13	1
LDPE_5% ZnO/V_CH	286	175	63	118	202	127	15	5

**Table 3:** The friction coefficients and abrasion depths of the LDPE samples measured for two condition modes, F2N_v5 and F1N_v1.

Samples/Test Names	Friction Coefficient (-)		Abrasion Depth (µm)
	F2N_v5	F1N_v1	F1N_v1
LDPE	0.454	0.507	10.7
LDPE_1% ZnO/V	0.465	0.480	9.7
LDPE_3% ZnO/V	0.494	0.478	9.3
LDPE_5% ZnO/V	0.508	0.490	9.5
LDPE_1% ZnO/V_CH	0.480	0.560	13.1
LDPE_3% ZnO/V_CH	0.479	0.470	14.1
LDPE_5% ZnO/V_CH	0.457	0.451	11.6

**Table 4:** The average values of the LDPE plate microhardness (HIT—indentation hardness; HV—Vickers microhardness; and hm—maximum impression depth). Standard deviations are provided in parentheses.

Samples	HIT	HV	hm
	(MPa)	(-)	(µm)
LDPE	20.9 (1.6)	2.0 (0.2)	37.4 (1.8)
LDPE_1% ZnO/V	27.1 (0.5)	2.6 (0.1)	31.8 (0.2)
LDPE_3% ZnO/V	26.6 (0.7)	2.5 (0.1)	32.0 (0.3)
LDPE_5% ZnO/V	25.3 (1.3)	2.4 (0.1)	32.9 (0.7)
LDPE_1% ZnO/V_CH	27.2 (0.6)	2.6 (0.1)	31.7 (0.3)
LDPE_3% ZnO/V_CH	16.7 (1.4)	1.6 (0.1)	41.9 (1.7)
LDPE_5% ZnO/V_CH	23.0 (1.3)	2.2 (0.1)	34.8 (0.9)
